# Risk of abnormal pregnancy outcomes after using ondansetron during pregnancy: A systematic review and meta-analysis

**DOI:** 10.3389/fphar.2022.951072

**Published:** 2022-09-02

**Authors:** Xiao Cao, Mingyao Sun, QiuYu Yang, Qi Wang, Liangying Hou, Jing Wang, Yu Wu, Long Ge

**Affiliations:** ^1^ Evidence-Based Nursing Centre, School of Nursing, Lanzhou University, Lanzhou, China; ^2^ Department of Social Medicine and Health Management, and Evidence Based Social Science Research Centre, School of Public Health, Lanzhou University, Lanzhou, China; ^3^ Department of Obstetrics and Gynecology, The First Hospital of Lanzhou University, Key Laboratory of Gynecologic Oncology of Gansu Province, Lanzhou, Gansu, China; ^4^ Key Laboratory of Evidence Based Medicine and Knowledge Translation of Gansu Province, Lanzhou, China

**Keywords:** ondansetron, pregnancy, abnormal pregnancy outcomes, defects, meta-analysis

## Abstract

**Background:** Hyperemesis gravidarum is a serious pregnancy complication that affects approximately 1% of pregnancies worldwide.

**Objective:** To determine whether the use of ondansetron during pregnancy is associated with abnormal pregnancy outcomes.

**Search strategy:** PubMed, Cochrane Library, CINAHL, Embase, CNKI, CBM, WANFANG, and ClinicalTrials.gov were searched for citations published in any language from inception to 15 December 2021.

**Selection criteria:** Eligible studies included any observational study.

**Data collection and analysis:** Odds ratio (OR) and 95% confidence interval (CI) were used as indicators to examine the association between ondansetron and abnormal pregnancy outcomes.

**Main results:** Twenty articles from 1,558 citations were included. Our preliminary analysis showed that compared with the unexposed group, the use of ondansetron during pregnancy may be associated with an increased incidence of cardiac defects (OR = 1.06, 95% CI: 1.01–1.10), neural tube defects (OR = 1.12, 95% CI: 1.05–1.18), and chest cleft (OR = 1.21, 95% CI: 1.07–1.37). Further sensitivity analysis showed no significant association between ondansetron and cardiac defects (OR = 1.15,95% CI: 0.94–1.40) or neural tube defects (OR = 0.87,95% CI: 0.46–1.66). When controversial studies were eliminated, the results for the chest defects disappeared. Simultaneously, we found that the use of ondansetron was associated with a reduced incidence of miscarriage (OR = 0.53, 95% CI: 0.31–0.89). Ondansetron was not associated with orofacial clefts (OR = 1.09,95% CI: 0.95–1.25), spinal limb defects (OR = 1.14,95% CI: 0.89–1.46), urinary tract deformities (OR = 1.06,95% CI: 0.97–1.15), any congenital malformations (OR = 1.03,95% CI: 0.98–1.09), stillbirth (OR = 0.97,95% CI: 0.83–1.15), preterm birth (OR = 1.22,95% CI: 0.80–1.85), neonatal asphyxia (OR = 1.05,95% CI: 0.72–1.54), or neonatal development (OR = 1.18,95% CI: 0.96–1.44) in our primary analysis.

**Conclusion:** In our analysis, using ondansetron during pregnancy was not associated with abnormal pregnancy outcomes. Although our study did not find sufficient evidence of ondansetron and adverse pregnancy outcomes, future studies including the exposure period and dose of ondansetron, as well as controlling for disease status, may be useful to truly elucidate the potential risks and benefits of ondansetron.

## Introduction

About 90% of pregnant women have symptoms of nausea and/or vomiting (Fejzo et al., 2019). Hyperemesis gravidarum (HG) is a severe pregnancy complication that affects about 1% of pregnancies worldwide (Einarson et al., 2013). The psychological and physical burden of pregnant women increases when suffering from HG (Fiaschi et al., 2018). Most women who experience serious nausea and vomiting in pregnancy (NVP)/HG need to use one or more drugs to control their symptoms (Lowe et al., 2020). According to clinical guidelines, ondansetron is widely used as a second-line treatment option for severe NVP (Shehmar et al., 2016; Taylor et al., 2017; Erick et al., 2018; Fiaschi et al., 2019).

Currently, ondansetron is increasingly used to treat NVP and HG worldwide. Between 2001 and 2014, the utilization rate of ondansetron increased from less than 1% to 13–25%, resulting in about 500,000 to 1 million women exposed to ondansetron in 4 million pregnancies in the US (Koren, 2014; Taylor et al., 2017; Parker et al., 2018). In Australia and New Zealand, physicians are 25% and 75% likely to use ondansetron when treating NVP and HG, respectively (Raymond, 2013). In Norway, 0.3% of pregnant women take ondansetron prescriptions, of which 76.9% are initially used in the first 3 months of pregnancy (van Gelder and Nordeng, 2021). In France, ondansetron prescriptions involved only 53 women (0.1%) between 2004 and 2017 in Haute-Garonne, contrary to other countries, like the US (Hurault-Delarue et al., 2021),

In November 2019, the European Medicines Agency (EMA) Pharmacovigilance Risk Assessment Committee (PRAC) released an updated comprehensive assessment report recommending that ondansetron should not be prescribed in the first 3 months of pregnancy (Aurobindo Pharma - Milpharm Ltd, 2022). A review about ondansetron in pregnancy revisited does not approve the epilogue of the EMA/PRAC assessment report and this part of the Summary of Product Characteristics (SmPC), and the regulators should consider eliminating this part from the SmPC (Damkier et al., 2021). Indeed, the safety of ondansetron has not concluded a decision now. Most studies have not detected that taking ondansetron in the early stages of pregnancy can lead to abnormal pregnancy outcomes in women (Lavecchia et al., 2018; Kaplan et al., 2019), but others have observed an increasing risk of hypoplastic left heart, diaphragmatic hernia, and respiratory system anomalies (Carstairs, 2016; Picot et al., 2020). The most recent meta-analysis was published in 2020, and its search ended in November 2019; therefore, this meta-analysis was not included in the five recently published studies (Huybrechts et al., 2020; Lemon et al., 2020; Dormuth et al., 2021; Sakran et al., 2021; Suarez et al., 2021). Three of these five studies were large-scale studies, which included 456, 963/33, 677/1, 880, and 594 pregnant women exposed to ondansetron. Three of the five studies focused on not only the risk of malformations but also other abnormal pregnancy outcomes (Dormuth et al., 2021; Sakran et al., 2021; Suarez et al., 2021).

Therefore, this systematic review and meta-analysis aimed to explore the association between ondansetron exposure during pregnancy and abnormal pregnancy outcomes.

## Materials and methods

### Search strategy

PubMed, Cochrane Library, CINAHL, Embase, CNKI, CBM, WANFANG, and ClinicalTrials.gov were searched for citations published in any language from inception to 15 December 2021, including topics of ondansetron and pregnancy (for a complete retrieval strategy, Supplementary Table S1). In addition, we searched the references included in the research and related systematic reviews. There were no requirements for language or publishing forms.

### Eligibility criteria and study selection

Studies that met the following criteria were considered qualified: 1) population were pregnant women; 2) included the ondansetron group; 3) included healthy or disease-matched controls (gestational nausea and vomiting or gestational hyperemesis); 4) outcomes included the risk of abnormal pregnancy (such as stillbirth, preterm birth, and congenital malformations); and 5) included studies were observational studies (such as prospective cohort, nested case-control, case-control, or case-cohort designs). If an overlap was detected between the two studies, we tended to select studies with high methodological quality. Animal studies, editorials, and reviews were excluded. An inspector screened the titles and abstracts of all the retrieved studies. Studies that met the criteria were independently reviewed by another reviewer, and inconsistencies were resolved through discussion; if necessary, senior authors were consulted to reach a consensus.

### Data extraction

Two authors (XC and MYS) separately extracted information from the selected studies including the country, study design, participant characteristics, exposure factors, result evaluation, and statistical analysis (including adjustment for confounding factors), and the differences were discussed and resolved. We chose risk estimates with the most complete adjustment for confounding factors and their 95% confidence intervals. The authors were contacted for additional data, when necessary.

### Risk of bias of an individual study

Based on the selection and comparability of groups and the method of determining exposure or results, two reviewers (XC and MYS) independently assessed the risk of bias in the cohort and case-control studies using a revised version of the Newcastle–Ottawa scale (Wells et al., 2001; Stang, 2010). We judged methodological quality based on the total score: ≤5 considered low, 6–7 considered moderate, and 8–9 deemed high quality (Chaudhry et al., 2022). The reviewers were not blind to the author’s name, organization, achievements, or journals of the publication. Any differences were resolved by another author (QYY).

### Data synthesis and statistical analysis

Odds ratio (OR) and 95% confidence interval (CI) were used to determine the correlation between ondansetron and abnormal pregnancy outcomes, although hazard ratios (HRs) and relative risks (RRs) were used in the included studies. We used the inverse variance weighting method of random effect to calculate OR and 95% CI together (Ge et al., 2019). To confirm the specific type of outcomes, we classified the abnormal pregnancy outcomes. We used Cochrane’s Q test and I^2^ value to test the statistical heterogeneity in the study (Higgins et al., 2003). Begg’s rank correlation test was used to evaluate publication bias at a significant level of *p* < 0.05 when there were at least 10 studies (Begg and Mazumdar, 1994). We also conducted a sensitivity analysis through studies that excluded a controversial or high risk of bias. All analyses were performed using Review Manager 5.4.1 (Cochrane Cooperative in Copenhagen, Denmark) and Stata V.16.1 software (Stata Corp, College Station, Texas, United States).

## Results

### Study selection and characteristics

**FIGURE 1:**
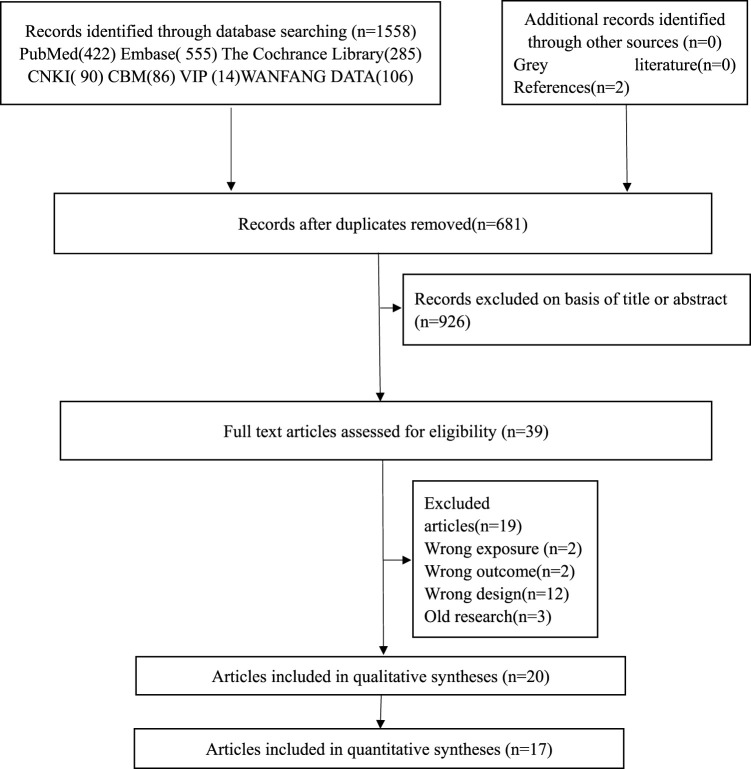
PRISMA flow diagram.

Literature screening and inclusion process were carried out according to PRISMA (Moher et al., 2009). Figure 1 shows the PRISMA flowchart. Our search searched 1558 records of which 39 were judged as possible studies based on the titles and abstracts (reasons and lists of references excluded from full-text filtering are presented in Supplementary Table S2) and 20 studies were eligible (Einarson et al., 2004; Asker et al., 2005; Anderka et al., 2012; Ferreira et al., 2012; Colvin et al., 2013; Pasternak et al., 2013; Danielsson et al., 2014; Werler et al., 2014; Özdemirci et al., 2014; Fejzo et al., 2016; Lemon et al., 2016; Parker et al., 2018; Bérard et al., 2019; Zambelli-Weiner et al., 2019; Couse and Syed, 2020; Huybrechts et al., 2020; Lemon et al., 2020; Dormuth et al., 2021; Sakran et al., 2021; Suarez et al., 2021). There were 11 cohort studies (Einarson et al., 2004; Asker et al., 2005; Colvin et al., 2013; Pasternak et al., 2013; Özdemirci et al., 2014; Fejzo et al., 2016; Bérard et al., 2019; Huybrechts et al., 2020; Lemon et al., 2020; Dormuth et al., 2021; Suarez et al., 2021), five case-control studies (Anderka et al., 2012; Danielsson et al., 2014; Werler et al., 2014; Parker et al., 2018; Zambelli-Weiner et al., 2019), two case reports (Lemon et al., 2016; Couse and Syed, 2020), and one study (Ferreira et al., 2012).

Eleven of the included studies originated from the United States, three from Canada, two from Sweden, and others apart from Turkey, Australia, Canada, Denmark, and Israel. The included studies contained a variety of controls, including chlorpromazine, unexposed, other anti-emetics, metoclopramide, non-teratogenic exposure (NTE), meclizine, and other prescription antiemetics (RxAE). Colvin et al., Berard et al., Asker et al., Dormuth et al., Lemon et al., Huybrechts et al., Suarez et al., Pasternak et al., Werler et al., Weiner et al. had one control group, unexposed, for comparison. ÖZDEMİRCİ et al. had one control group, chlorpromazine, for comparison. Fejzo et al. and Einarson et al. used two control groups for comparison, a disease matching group and an unexposed group. Parker et al. and Anderka et al. used two control groups for comparison, RxAE and unexposed. Sakran et al. observed the risk of defects in the ondansetron exposure group and the metoclopramide exposure group in the same cohort. Danielsson et al. detected the risk of the ondansetron exposure group and the meclizine exposure pregnancies. There were also two case report studies and one case series study. Table 1 presents the characteristics of the 20 studies. Supplementary Table S3 lists the adjustment variables of the included studies.

**TABLE 1:** Study characteristics.

Study	Study design	Country	The name of the cohort or data source	Study period	Exposure of the comparator group (disease status)	Exposition period	Sample size (exposed/unexposed) or (case/control)	Funding
Fejzo 2016 [33]	Cohort study	United States	The Hyperemesis Education and Research Foundation	2007–2014	Women with or without a history of HG^a^	NR	1070/771	Yes
ÖZDEMİRC 2014 [34]	Cohort study	Turkey	NR	2006–2011	Chlorpromazine	NR	100/85	NR
Colvin 2013 [35]	Cohort study	Australia	NR	2002–2005	Non-ondansetron	NR	251/96447	Yes
Berard 2019 [36]	Cohort study	Canada	The Quebec Pregnancy Cohort	1998–2015	Unexposed	The first trimester of pregnancy	31/224845	Yes
Asker 2005 [37]	Cohort study	Sweden	The Swedish Medical Birth Registry	1995–2002	Unexposed	First to third trimester	29804/665572	Yes
Dormuth 2021 [22]	Cohort study	Canada\USA\United Kingdom	Five Canadian provinces, the IBM Market Scan Research Databases from the US, and United Kingdom CPRD	2002–2016	Unexposed	The first 84 days of gestation	185086/3927936	Yes
Lemon 2020 [23]	Cohort study	United States	Magee–Womens Hospital of the University of Pittsburgh Medical Center (UPMC)	2006–2014	No ondansetron	The first trimester	3733/29944	Yes
Einarson 2004 [38]	Cohort study	Canada	Teratogen Information Services (TIS)	NR	Other antiemetics/Non-teratogen	NR	176/176/176	Yes
Huybrechts 2020 [24]	Cohort study	United States	The Nationwide Medicaid Analytic eXtract (MAX)	2000–2014	Unexposed	NR	23877/1856717	Yes
Suarez 2020 [25]	Cohort study	United States	The University of North Carolina (UNC) Health Care system	2014–2017	Comparators	NR	1742/935	Yes
Pasternak 2013 [39]	Cohort study	Denmark	The Medical Birth Registry and the National Patient Register in Denmark	2004–2011	Unexposed	The first trimester	1849/7396	Yes
Werler 2014 [40]	Case-control study	United States	NR	2007–2011	Controls	NR	646/2037	No
Weiner 2019 [41]	Case-control study	United States	US administrative health care database, the Truven Health Market Scan Commercial Database	2000–2014	Unexposed During Pregnancy	The first trimester	76330/787753	Yes
Sakran 2021 [21]	Case-control study	Israeli	The Israeli Teratology Information Service	2010–2014	Metoclopramide/NTE	NR	195/888	NR
Danielsson 2014 [42]	Case–control study	Sweden	The Swedish Medical Birth Register combined with the Swedish Register of Prescribed Drugs	1998–2012	Meclozine	10–12w	1349/1500085	NR
Parker 2018 [43]	Case–control study	United States	The National Birth Defects Prevention Study/the Slone Birth Defects Study^b^	2005–2011/1997–2014	RxAE/No Treatment	The first trimester	253/6498; 375/5498	Yes
Anderka 2013 [44]	Case–control study	United States	The National Birth Defects Prevention Study	1997–2004	RxAE	The first trimester	621/4021	NR
Couse 2020 [45]	Case report study	United States	NR	NR	NR	NR	NR	No
Lemon 2016 [46]	Case report study	United States	NR	NR	NR	NR	NR	Yes
Ferreira 2012 [47]	Case series study	United States	NR	2002–2011	NR	NR	14	NR

a: this study contained two comparison groups and was therefore considered two studies in the data analysis; b: this study contained two different studies and was therefore considered two studies in the data analysis.

## Methodological quality of an individual study

Only two of the eligible studies were categorized as high methodological quality, most (13/16) of the eligible studies were categorized as moderate methodological quality, and only one (5%) low-quality study was received by Einarson et al. (Einarson et al., 2004). Three cohort studies (Einarson et al., 2004; Colvin et al., 2013; Fejzo et al., 2016) and four case-control studies (Werler et al., 2014; Parker et al., 2018; Zambelli-Weiner et al., 2019) had a high risk of bias in assessing exposure since they did not mention any information or reference about the effectiveness of ondansetron measurements or only baseline measurements. Ten cohort studies are at high risk of bias for comparability of cohorts based on the design or analysis (Einarson et al., 2004; Asker et al., 2005; Colvin et al., 2013; Pasternak et al., 2013; Özdemirci et al., 2014; Bérard et al., 2019; Huybrechts et al., 2020; Lemon et al., 2020; Dormuth et al., 2021; Suarez et al., 2021), and four case-control studies (Anderka et al., 2012; Danielsson et al., 2014; Werler et al., 2014; Parker et al., 2018) had a high risk of bias for study controls for any additional factor. Four (36.36%) cohort studies (Einarson et al., 2004; Asker et al., 2005; Özdemirci et al., 2014; Fejzo et al., 2016) were at high risk of bias for outcome assessment. One cohort study (Suarez et al., 2021) was at high risk of bias for adequacy of follow-up of cohorts. Two case-report studies and one case-series study did not assess the risk of bias. Supplementary Figure S3 showed the risk of bias in the included studies.

Although the methodological quality of the Weiner study was high according to the NOS results, the European Network of Teratology Information Services (ENTIS) Scientific Committee’s position was that the study was compromised to a certain extent methodologically and ethically and that the results could not be considered when evaluating the totality of evidence on ondansetron safety in pregnancy (Colvin et al., 2013). Therefore, our study considered Weiner's study to be a controversial study, which was dealt with in sensitivity analysis.

### Birth defects

#### Cardiac defects

**FIGURE 2:**
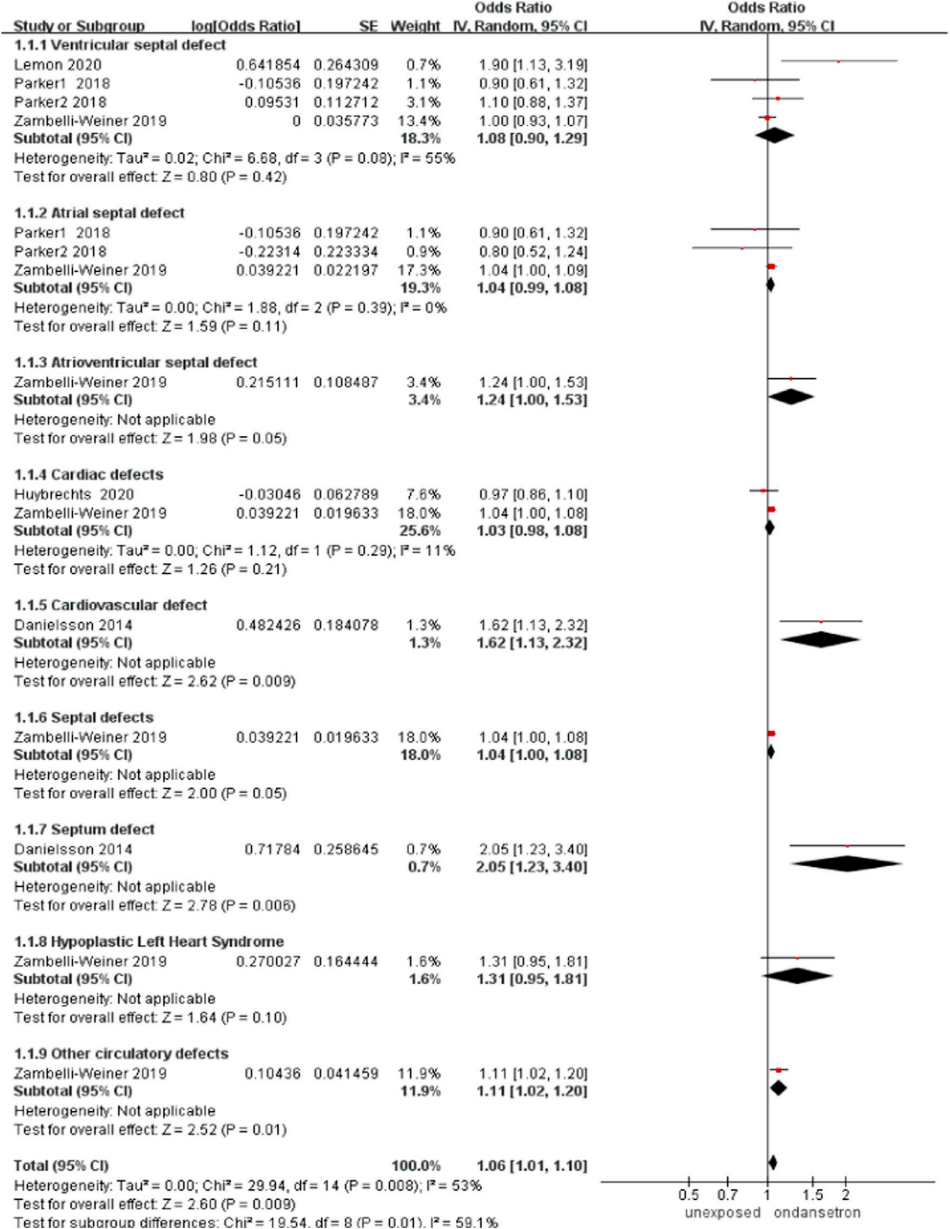
Forest plot of cardiac defects.

In this analysis, five studies detected a total of 104,763 infants exposed to ondansetron and 2687298 control infants reported cardiac defects (Parker et al., 2018; Zambelli-Weiner et al., 2019; Huybrechts et al., 2020; Lemon et al., 2020; Sakran et al., 2021). The incidence of cardiac defects increased significantly after the use of ondansetron during pregnancy (OR = 1.06,95% CI: 1.01–1.10) in our primary analysis (Figure 2). To confirm the types of cardiac defects, we conducted subgroup analyses of different types of cardiac defects. Further research showed a significant increase in the rate of cardiovascular defects (OR = 1.62, 95% CI: 1.13–2.32), septum defects (OR = 2.05, 95% CI: 1.23–3.40), and other circulatory defects (OR = 1.11, 95% CI: 1.02–1.20) was detected following ondansetron use during pregnancy. Further sensitivity analysis showed no increase in heart defects (OR = 1.15, 95% CI: 0.94–1.40, Supplementary Figure S1). The included case-report studies reported the outcomes of isolated atrial and ventricular septal defects.

### Orofacial clefts

**FIGURE 3:**
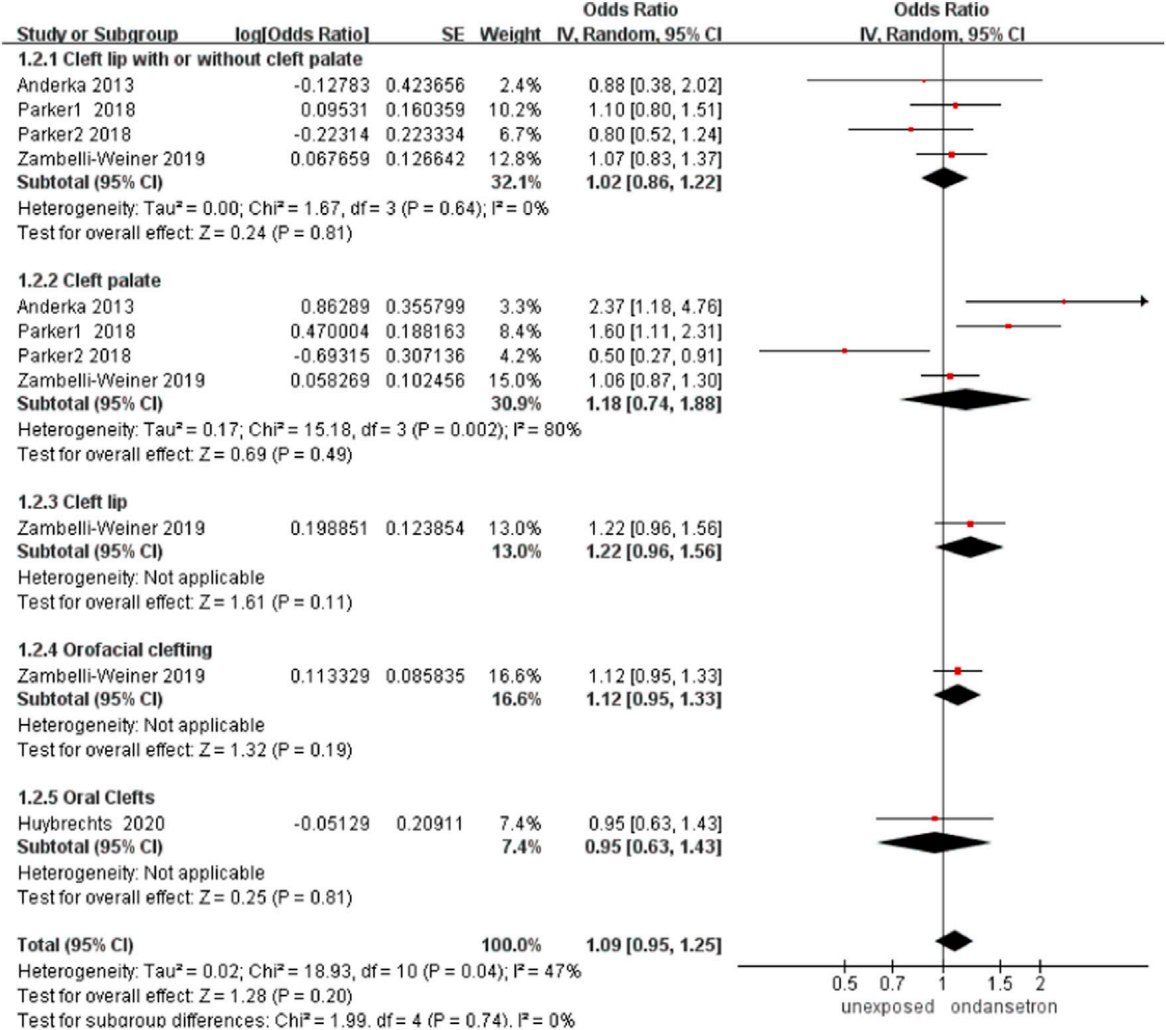
Forest plot of orofacial clefts.

In this analysis, a total of 101,459 infants exposed to ondansetron and 2,660,487 control infants reported orofacial clefts in four studies (Anderka et al., 2012; Parker et al., 2018; Zambelli-Weiner et al., 2019; Huybrechts et al., 2020). The orofacial cleft rate did not increase significantly after the use of ondansetron during pregnancy (OR = 1.09, 95% CI: 0.95–1.25) in our primary analysis (Figure 3). Sensitivity analysis by excluding controversial studies showed the same result that no risk of orofacial clefts was detected (OR = 1.05, 95% CI: 0.77–1.44, Supplementary Figure S2).

### Spinal limb defects

**FIGURE 4:**
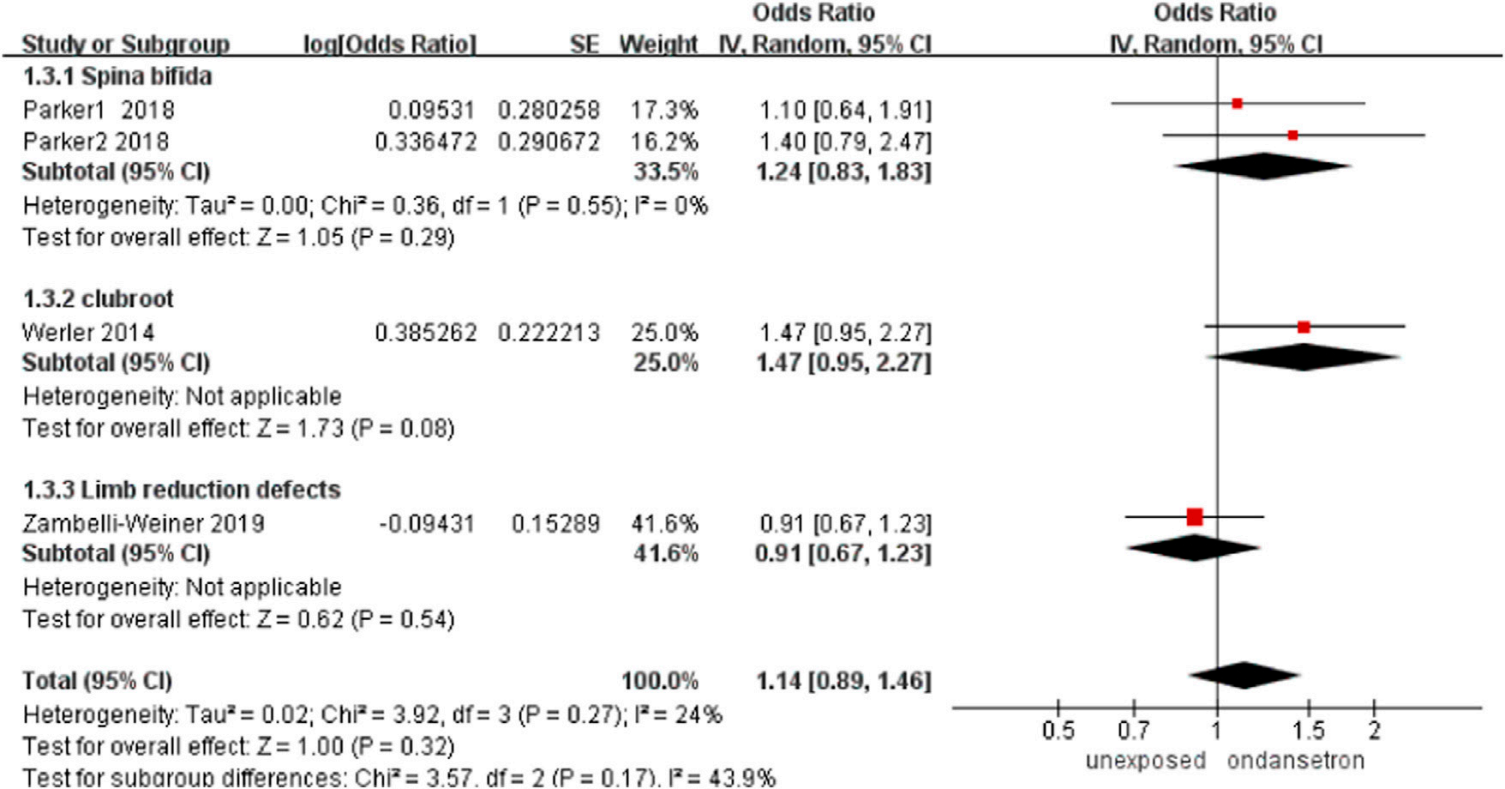
Forest plot of spinal limb defects.

In this analysis, a total of 77,604 infants exposed to ondansetron and 801,247 control infants reported spinal limb defects (Werler et al., 2014; Parker et al., 2018; Zambelli-Weiner et al., 2019). There was no obvious increase in the incidence of spinal limb defects after administration of ondansetron during pregnancy (OR = 1.14, 95% CI: 0.89–1.46) in our primary analysis (Figure 4). Sensitivity analysis by excluding controversial studies showed the same result that no risk of spinal limb defects was detected (OR = 1.34, 95% CI: 1.00–1.79, Supplementary Figure S3).

### Neural tube defects

**FIGURE 5:**
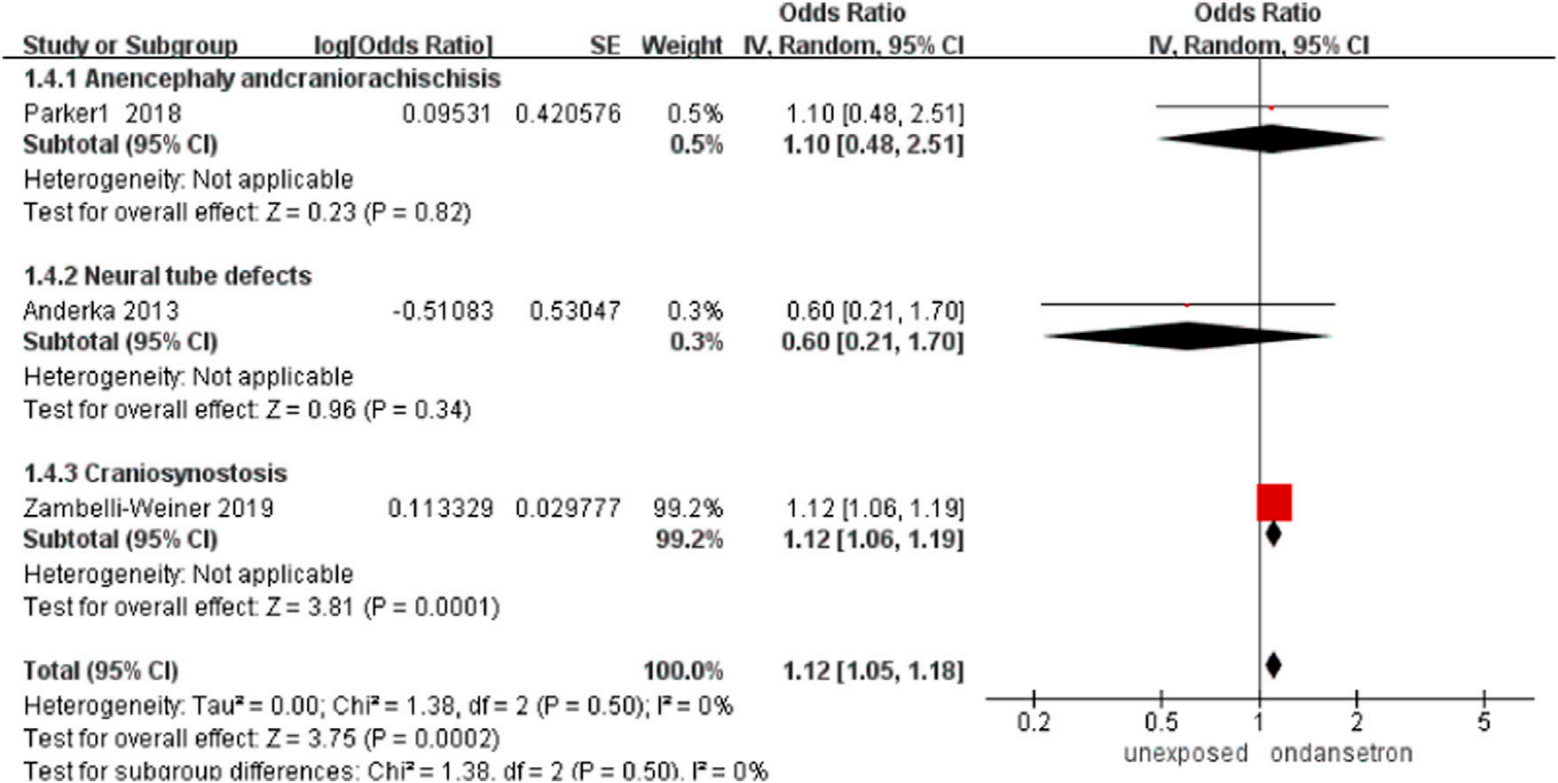
Forest plot of neural tube defects.

In this analysis, a total of 77,579 infants exposed to ondansetron and 803,231 control infants reported neural tube defects (Anderka et al., 2012; Parker et al., 2018; Zambelli-Weiner et al., 2019). The rate of neural tube defects increased significantly after using ondansetron during pregnancy (OR = 1.12, 95% CI: 1.05–1.18) in our primary analysis (Figure 5). Sensitivity analysis by excluding controversial studies showed a different result that no risk of neural tube defects was detected (OR = 0.87, 95% CI: 0.46–1.66, Supplementary Figure S4).

### Urinary tract deformities

**FIGURE 6:**
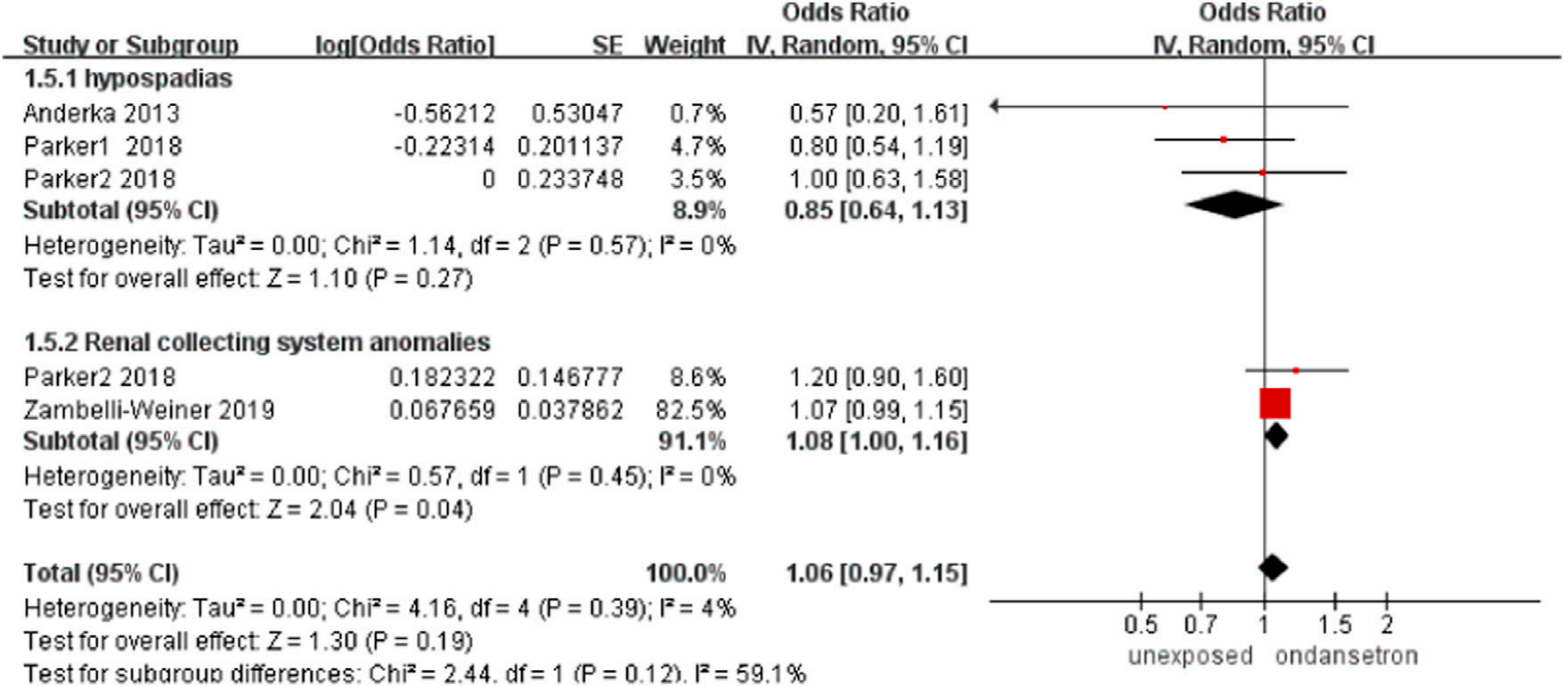
Forest plot of urinary tract deformities.

In this analysis, 77,579 infants exposed to ondansetron and 803,231 control infants reported urinary tract deformities (Anderka et al., 2012; Parker et al., 2018; Zambelli-Weiner et al., 2019). The incidence of urinary tract deformities did not increase significantly after ondansetron was used during pregnancy (OR = 1.06, 95% CI: 0.97–1.15) in our primary analysis (Figure 6). Sensitivity analysis by excluding controversial studies showed the same result that no risk of urinary tract deformities was detected (OR = 0.99, 95% CI: 0.77–1.26, Supplementary Figure S5).

#### Chest defects

**FIGURE 7:**
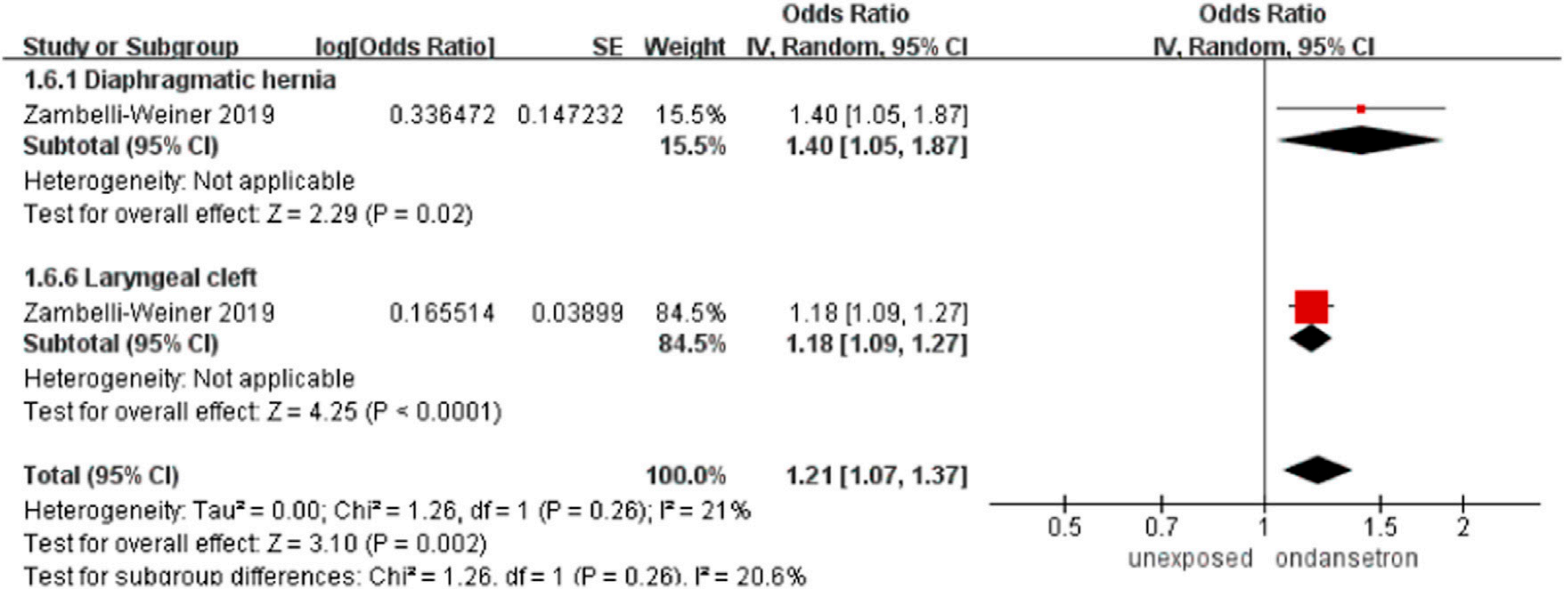
Forest plot of chest defects.

One study detected a total number of 76,330 infants exposed to ondansetron and 787,753 control infants reported chest defects in this analysis (Zambelli-Weiner et al., 2019). The incidence of chest defects increased significantly after the use of ondansetron during pregnancy (OR = 1.21, 95% CI: 1.07–1.37) in our primary analysis (Figure 7). It is important to note that the results on chest defects were obtained from Weiner’s study. If we ruled out controversial studies, then the association between ondansetron and chest defects disappeared.

### Any congenital malformation

**FIGURE 8:**
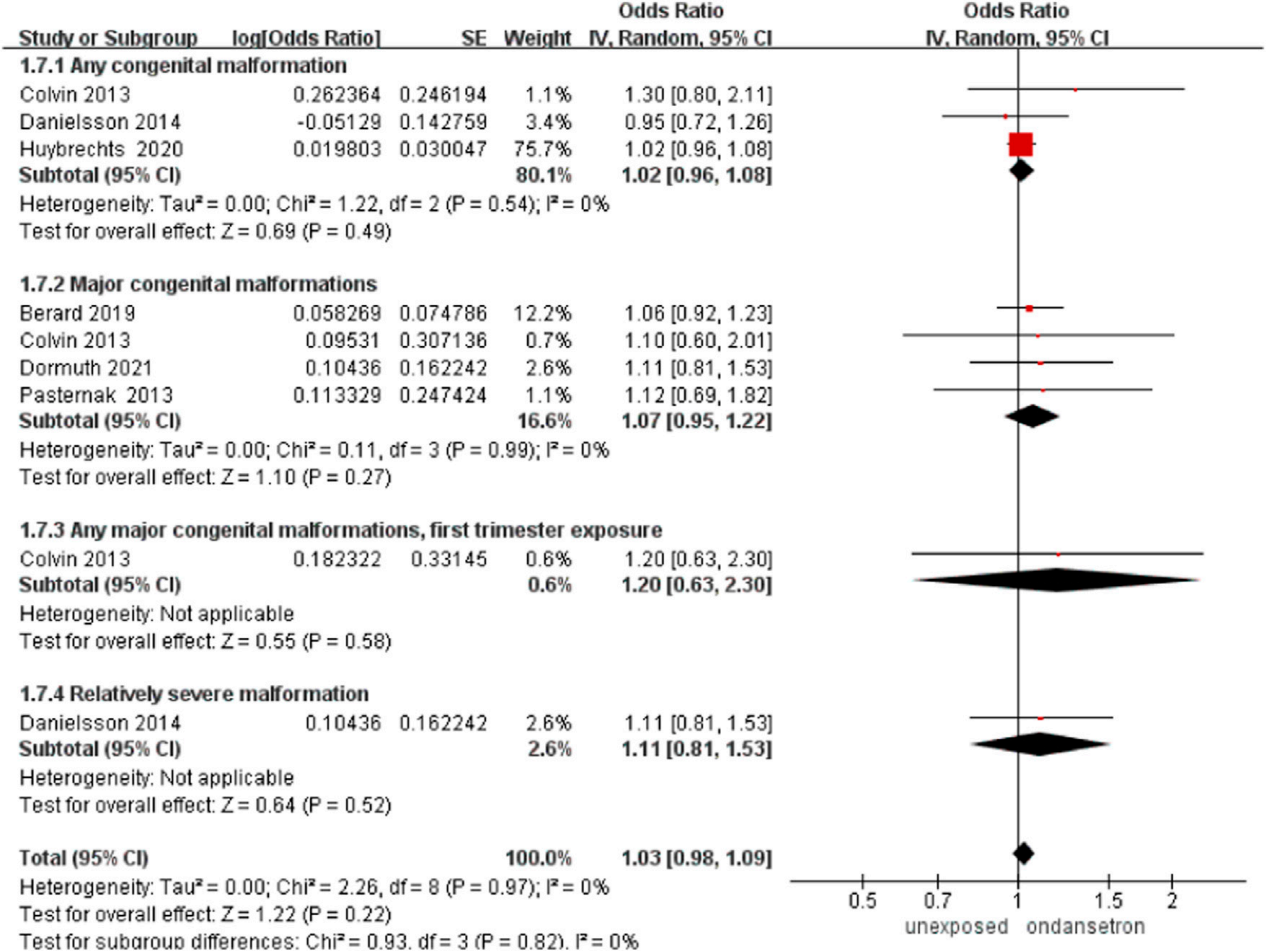
Forest plot of any congenital malformations.

Six studies detected 212,443 infants exposed to ondansetron, and 7,613,426 control infants reported any congenital malformations in the analysis (Colvin et al., 2013; Pasternak et al., 2013; Danielsson et al., 2014; Bérard et al., 2019; Huybrechts et al., 2020; Dormuth et al., 2021). Any congenital malformation data were derived directly from the corresponding classification in the included studies. No obvious increase in congenital malformations was found after administration of ondansetron during pregnancy (OR = 1.03, 95% CI: 0.98–1.09) in our primary analysis (Figure 8). This outcome did not include any studies that required exclusion.

### Other defects (negative control)

One study detected 76,330 ondansetron-exposed and 787,753 control infants reported other defects (negative control) (Zambelli-Weiner et al., 2019). The rate of other defects (negative control) did not increase significantly after ondansetron was used during pregnancy (OR = 1.02, 95% CI: 1.00–1.04).

## Adverse Fetal outcomes

### Miscarriage

**FIGURE 9:**
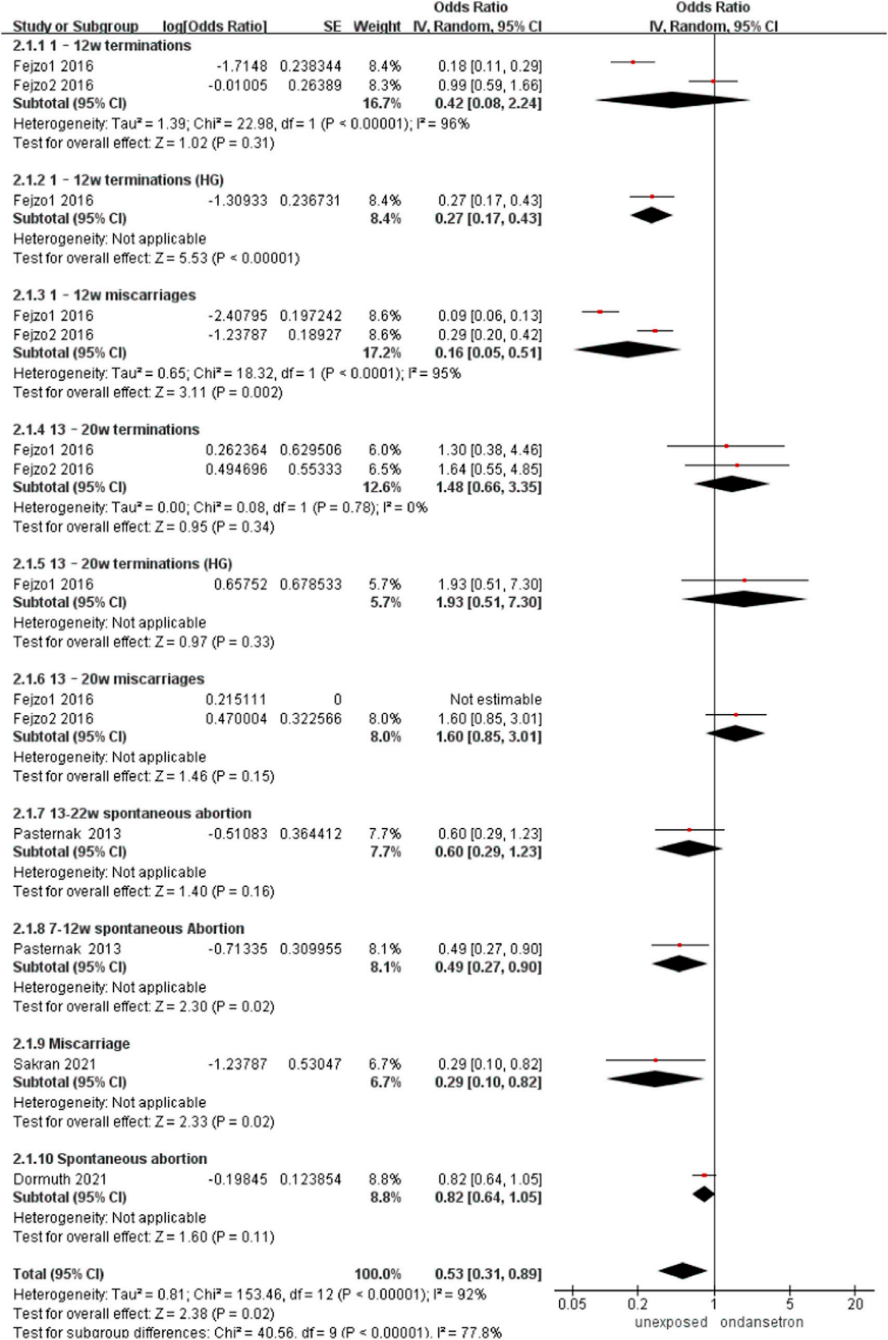
Forest plot of miscarriage.

Four studies detected 188,200 ondansetron-exposed, and 3,936,991 control infants reported miscarriage (Pasternak et al., 2013; Fejzo et al., 2016; Dormuth et al., 2021; Sakran et al., 2021). Use of ondansetron during pregnancy can significantly reduce the rate of miscarriage (OR = 0.53, 95% CI: 0.31–0.89) in our primary analysis (Figure 9). This outcome did not include any studies that needed to be excluded in sensitivity analysis.

### Stillbirth

**FIGURE 10:**
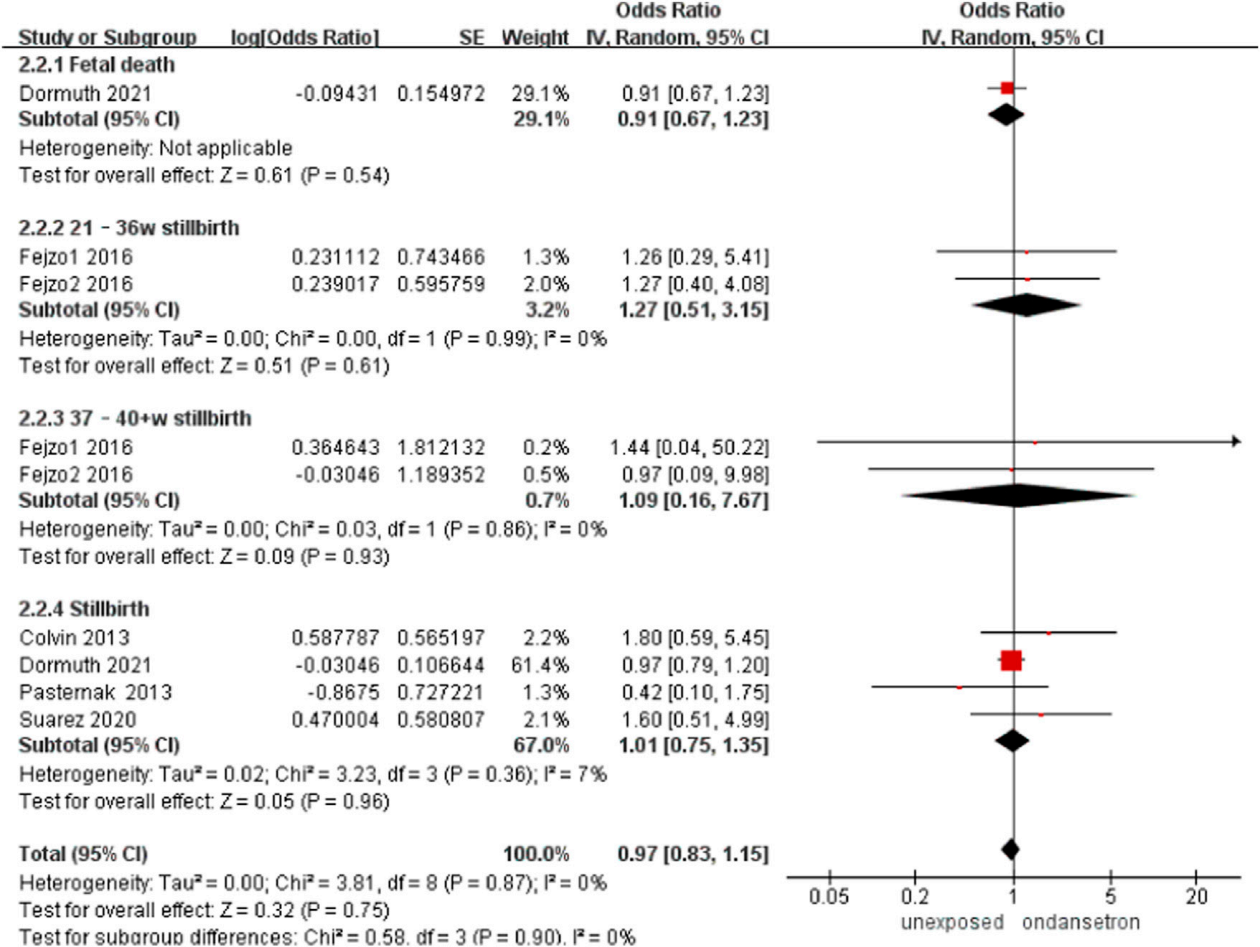
Forest plot of stillbirth.

Five studies detected 189,998 ondansetron-exposed, and 4,033,485 control infants reported stillbirth.^22,25,33,35,39^ There was no significant change in stillbirth rate after administration of ondansetron during pregnancy (OR = 0.97, 95% CI: 0.83–1.15) in our primary analysis (Figure 10). This outcome did not include any studies that needed to be excluded in sensitivity analysis.

### Preterm birth

**FIGURE 11:**
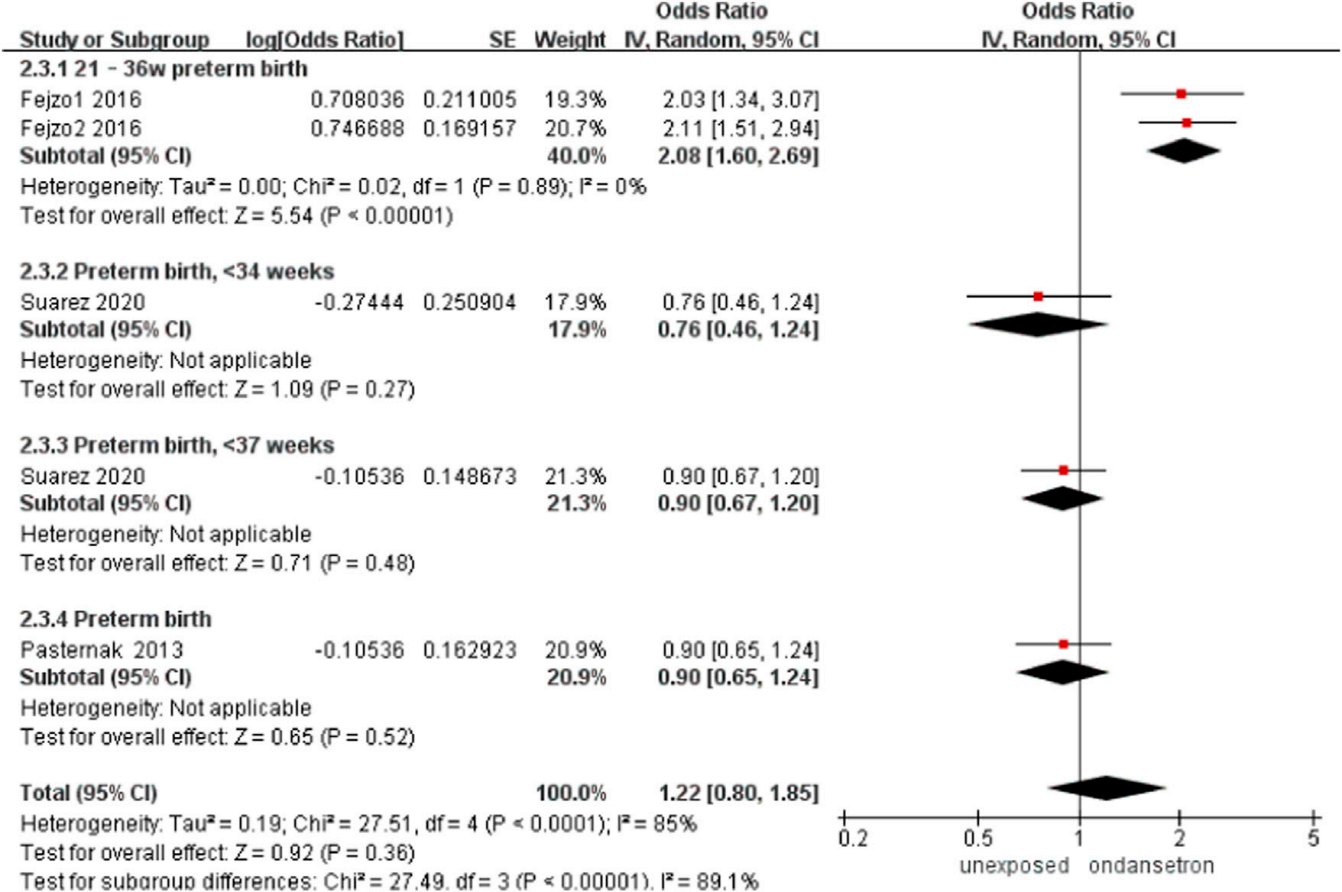
Forest plot of preterm birth.

Three studies detected 4,661 ondansetron-exposed and 9,102 control infants reported preterm birth (Pasternak et al., 2013; Fejzo et al., 2016; Suarez et al., 2021). No change in the incidence of preterm birth was found after administration of ondansetron in pregnancy (OR = 1.22, 95% CI: 0.80–1.85) in our primary analysis (Figure 11). Sensitivity analysis was not required for this part.

### Neonatal asphyxia

**FIGURE 12:**
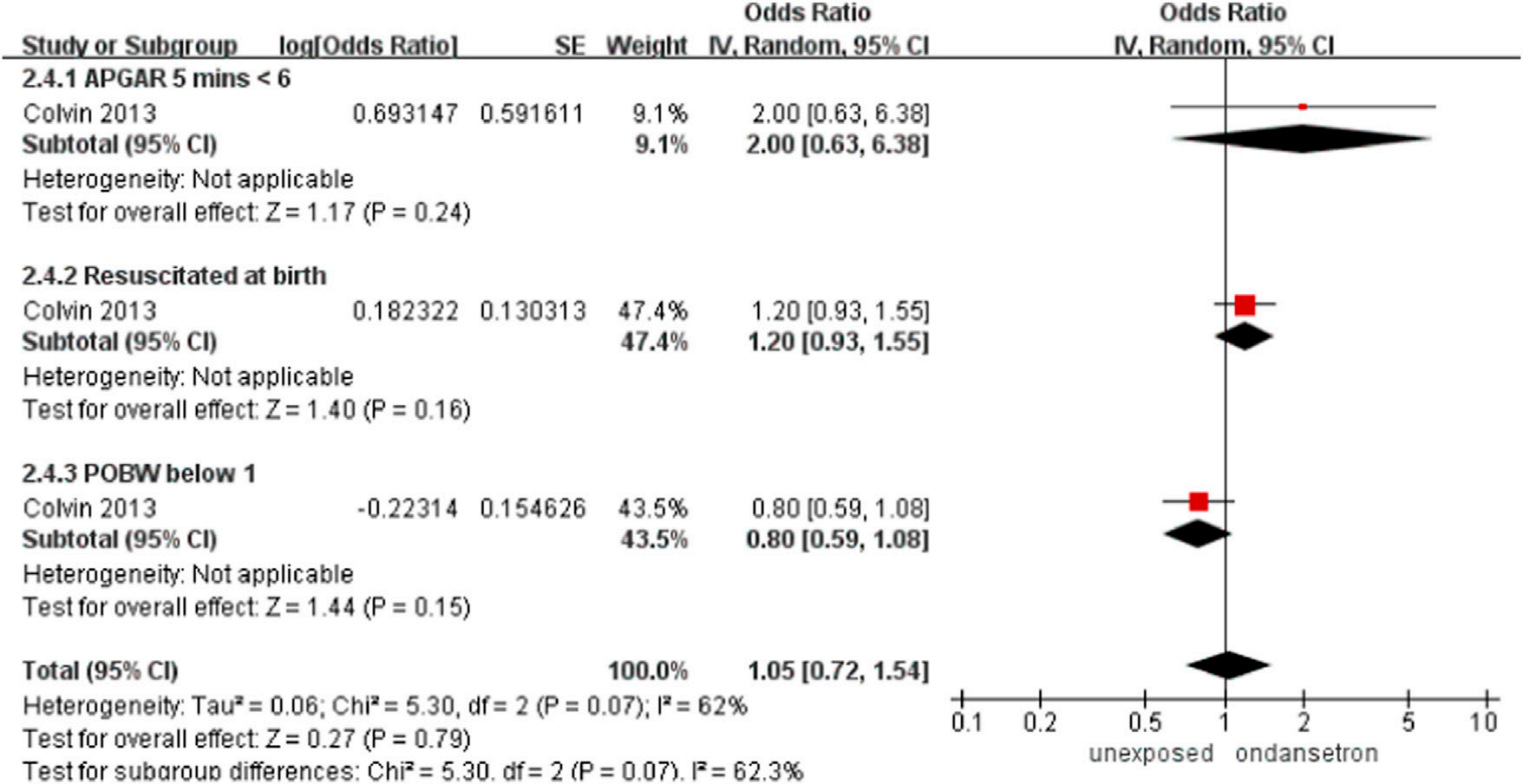
Forest plot of neonatal asphyxia.

One study detected 251 ondansetron-exposed, and 96,447 control infants reported neonatal asphyxia (Colvin et al., 2013). There was no obvious change in the incidence of neonatal asphyxia after administration of ondansetron in pregnancy (OR = 1.05, 95% CI: 0.72–1.54). (Figure 12).

### Neonatal development

**FIGURE 13:**
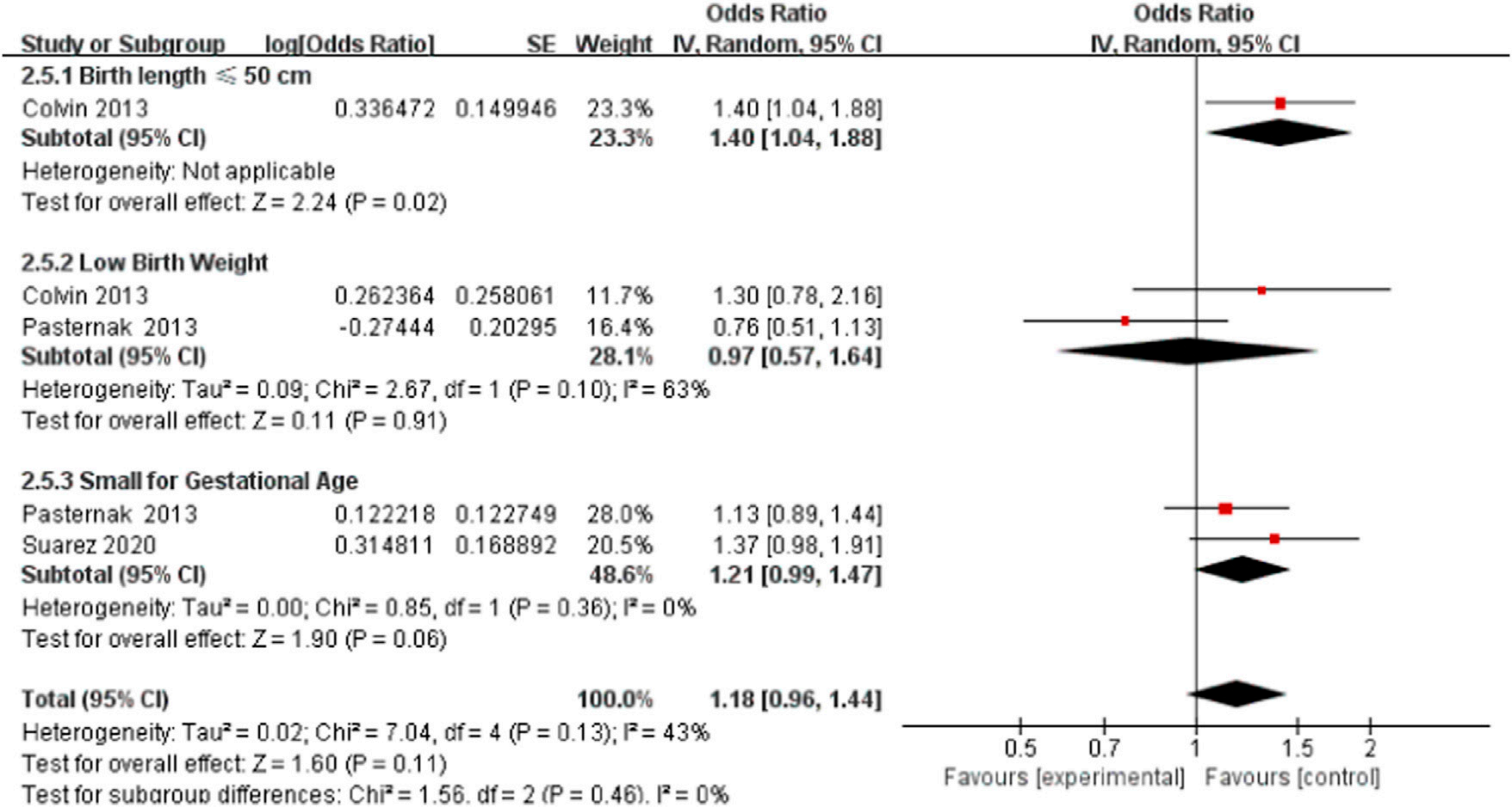
Forest plot of neonatal development.

Three studies detected 3,842 ondansetron-exposed, and 104,778 control infants reported neonatal development (Colvin et al., 2013; Pasternak et al., 2013; Suarez et al., 2021). The indicators of abnormal neonatal development included low birth weight, small gestational age, and low birth length. After the use of ondansetron during pregnancy, there was no change in the risk of abnormal neonatal development (OR = 1.18, 95% CI: 0.96–1.44) in our primary analysis (Figure 13).

The included case-report studies reported the outcomes included pharmacokinetic changes, intrauterine growth retardation, transient tachypnea, a mild hydrocele, and extrarenal pelvis.

## Discussion

Our findings enrich the previous meta-analysis to observe whether or not there is an association between ondansetron use and risk of abnormal pregnancy outcomes, which has not been fully addressed previously. This study of 20 observational studies showed that ondansetron users were at a statistically higher risk of cardiac defects, neural tube defects, and chest defects than unexposed individuals, while the risk of miscarriage was statistically lower. Based on Damkier’s comment, we excluded Weiner’s study (Damkier et al., 2021). As a result, we did not observe a correlation between the use of ondansetron and any abnormal pregnancy outcomes.

Previously published systematic reviews have focused on the association between ondansetron use and the occurrence of congenital malformations, and there are inconsistencies in their conclusions (Carstairs, 2016; Lavecchia et al., 2018; Kaplan et al., 2019; Picot et al., 2020). Kaplan et al. (2019) showed that there was no exact incidence of cardiac defects, orofacial clefts, major malformations, hypospadias, or genitourinary malformations. Picot et al. (2020) found that exposure to ondansetron in early pregnancy was associated with an increased incidence of the ventricular septal defect (OR 1.11, 95% CI 1.00–1.23) and oral clefts (OR 1.22, 95% CI 1.00–1.49). Lavecchia et al. (2018) found that the increase in the incidence of specific defects, such as cardiovascular defects and cleft palate, was contradictory. Carstairs’s analysis found that the incidence of birth defects related to ondansetron exposure appeared to be low and that the incidence of heart abnormalities in newborns exposed to ondansetron might increase Carstairs (2016). In previous studies on the use of ondansetron during pregnancy, a very major issue was the resulting risk of heart defects, cleft palate, and other malformations. Our meta-analysis of observational studies detected that those who took ondansetron during pregnancy did not have an increased risk of overall heart defect or cleft palate when compared with the control group.

The prescription of unlabeled ondansetron has increased sharply in pregnant women (Colvin et al., 2013). Hyperemesis gravidarum that does not respond to other treatments may be treated with ondansetron.

## Strengths and limitations

Our review included a relatively large number of studies (20) and a large number of participants from nine countries (9,445,268). This study included cohort and case-control studies, also taking into account case reports. Simultaneously, we used the adjusted effect value, and the result was closer to the real effect. Most of the results included in the studies were adjusted; therefore, there was less likelihood that confounding factors would affect the credibility of the results.

Our study had some limitations. For some outcomes, the small number of participants included in the study may have made the findings unreliable. At the same time, our systematic review did not process OR/RR/HR conversion; therefore, the aggregate values may be biased. However, most studies had unclear or wide-ranging exposure periods and doses; therefore, it was not possible to judge whether ondansetron was used in the exposure group during fetal organ development and the teratogenic dose. Moreover, most studies did not control for disease status (NVP). Ondansetron is generally prescribed for severe NVP (HG), which may be a confounding factor because it is associated with poor maternal, fetal, and child outcomes (Fejzo et al., 2019).

## Conclusions and implications for future research

In conclusion, we found that there was no sufficient evidence to construct the association between ondansetron and adverse pregnancy outcomes. Our findings did not support the conclusions of the EMA/PRAC that recommended against the use of ondansetron in early pregnancy. Future studies should focus on the exposure period and dose of ondansetron, as well as controlling for disease status to truly elucidate the potential risks and benefits of ondansetron.

## Data Availability

The original contributions presented in the study are included in the article/Supplementary Material; further inquiries can be directed to the corresponding authors.

## References

[B1] Anderka M. Mitchell A. A. Louik C. Werler M. M. Hernandez-Diaz S. Rasmussen S. A. (2012). Medications used to treat nausea and vomiting of pregnancy and the risk of selected birth defects. Birth Defects Res. A Clin. Mol. Teratol. 94 (1), 22–30. 10.1002/bdra.22865 22102545 PMC3299087

[B2] Asker C. Norstedt Wikner B. Källén B. (2005). Use of antiemetic drugs during pregnancy in Sweden. Eur. J. Clin. Pharmacol. 61 (12), 899–906. 10.1007/s00228-005-0055-1 16328314

[B3] Aurobindo Pharma - Milpharm Ltd (2022) Ondansetron 4 mg film-coated tablets - summary of product characteristics (SmPC) - (emc). Available at: https://www.medicines.org.uk/emc/medicine/32781/spc .

[B4] Begg C. B. Mazumdar M. (1994). Operating characteristics of a rank correlation test for publication bias. Biometrics 50 (4), 1088. PMID: 7786990. 10.2307/2533446 7786990

[B5] Bérard A. Sheehy O. Gorgui J. Zhao J. P. Soares de Moura C. Bernatsky S. (2019). New evidence for concern over the risk of birth defects from medications for nausea and vomitting of pregnancy. J. Clin. Epidemiol. 116, 39–48. 10.1016/j.jclinepi.2019.07.014 31352006

[B6] Carstairs S. D. (2016). Ondansetron use in pregnancy and birth defects: A systematic review. Obstet. Gynecol. 127 (5), 878–883. 10.1097/AOG.0000000000001388 27054939

[B7] Chaudhry D. Chaudhry A. Peracha J. Sharif A. (2022). Survival for waitlisted kidney failure patients receiving transplantation versus remaining on waiting list: Systematic review and meta-analysis. BMJ 376, e068769. 10.1136/bmj-2021-068769 35232772 PMC8886447

[B8] Colvin L. Gill A. W. Slack-Smith L. Stanley F. J. Bower C. (2013). Off-label use of ondansetron in pregnancy in Western Australia. Biomed. Res. Int. 2013, 909860. 10.1155/2013/909860 24396830 PMC3874333

[B9] Couse M. Syed S. (2020). A rise in aspartate transaminase and alanine transaminase associated with ondansetron administration in a pregnant female. Cureus 12 (11), e11540. 10.7759/cureus.11540 33365209 PMC7748552

[B10] Damkier P. Kaplan Y. C. Shechtman S. Diav-Citrin O. Cassina M. Weber-Schoendorfer C. (2021). Ondansetron in pregnancy revisited: Assessment and pregnancy labelling by the European Medicines agency (EMA) & pharmacovigilance risk assessment committee (PRAC). Basic Clin. Pharmacol. Toxicol. 128 (4), 579–582. 10.1111/bcpt.13541 33275828

[B11] Danielsson B. Wikner B. N. Källén B. (2014). Use of ondansetron during pregnancy and congenital malformations in the infant. Reprod. Toxicol. 50, 134–137. 10.1016/j.reprotox.2014.10.017 25450422

[B12] Dormuth C. R. Winquist B. Fisher A. Wu F. Reynier P. Suissa S. (2021). Comparison of pregnancy outcomes of patients treated with ondansetron vs alternative antiemetic medications in a multinational, population-based cohort. JAMA Netw. Open 4 (4), e215329. 10.1001/jamanetworkopen.2021.5329 33890993 PMC8065380

[B13] Einarson A. Maltepe C. Navioz Y. Kennedy D. Tan M. P. Koren G. (2004). The safety of ondansetron for nausea and vomiting of pregnancy: A prospective comparative study. BJOG 111 (9), 940–943. 10.1111/j.1471-0528.2004.00236.x 15327608

[B14] Einarson T. R. Piwko C. Koren G. (2013). Quantifying the global rates of nausea and vomiting of pregnancy: A meta analysis. J. Popul. Ther. Clin. Pharmacol. 20 (2), e171–83. PMID: 23863575. 23863575

[B15] Erick M. Cox J. T. Mogensen K. M. (2018). ACOG practice bulletin 189: Nausea and vomiting of pregnancy. Obstet. Gynecol. 131 (5), 935. 10.1097/AOG.0000000000002604 29683896

[B16] Fejzo M. S. MacGibbon K. W. Mullin P. M. (2016). Ondansetron in pregnancy and risk of adverse fetal outcomes in the United States. Reprod. Toxicol. 62, 87–91. 10.1016/j.reprotox.2016.04.027 27151373

[B17] Fejzo M. S. Trovik J. Grooten I. J. Sridharan K. Roseboom T. J. Vikanes A. (2019). Nausea and vomiting of pregnancy and hyperemesis gravidarum. Nat. Rev. Dis. Prim. 5, 62–17. 10.1038/s41572-019-0110-3 31515515

[B18] Ferreira E. Gillet M. Lelièvre J. Bussieres J. F. (2012). Ondansetron use during pregnancy: A case series. J. Popul. Ther. Clin. Pharmacol. 19 (1), e1–e10. PMID: 22267256. 22267256

[B19] Fiaschi L. Nelson- Piercy C. Gibson J. Szatkowski L. Tata L. J. (2018). Adverse maternal and birth outcomes in women admitted to hospital for hyperemesis gravidarum: A population- based cohort study. Paediatr. Perinat. Epidemiol. 32, 40–51. 10.1111/ppe.12416 28984372

[B20] Fiaschi L. Nelson-Piercy C. Deb S. King R. Tata L. J. (2019). Clinical management of nausea and vomiting in pregnancy and hyperemesis gravidarum across primary and secondary care: A population-based study. BJOG 126 (10), 1201–1211. 10.1111/1471-0528.15662 30786126

[B21] Ge L. Guyatt G. Tian J. Pan B. Chang Y. Chen Y. (2019). Insomnia and risk of mortality from all-cause, cardiovascular disease, and cancer: Systematic review and meta-analysis of prospective cohort studies. Sleep. Med. Rev. 48, 101215. 10.1016/j.smrv.2019.101215 31630016

[B22] Higgins J. P. Thompson S. G. Deeks J. J. Altman D. G. (2003). Measuring inconsistency in meta-analyses. BMJ 327 (7414), 557–560. 10.1136/bmj.327.7414.557 12958120 PMC192859

[B23] Hurault-Delarue C. Araujo M. Vabre C. Benevent J. Damase-Michel C. Lacroix I. (2021). What changes in prescription patterns of antiemetic medications in pregnant women in France? Fundam. Clin. Pharmacol. 35 (6), 1159–1167. 10.1111/fcp.12681 33866614

[B24] Huybrechts K. F. Hernandez-Diaz S. Straub L. Gray K. J. Zhu Y. Mogun H. (2020). Intravenous ondansetron in pregnancy and risk of congenital malformations. JAMA 323 (4), 372–374. 10.1001/jama.2019.18587 31730152 PMC6865841

[B25] Kaplan Y. C. Richardson J. L. Keskin-Arslan E. Erol-Coskun H. Kennedy D. (2019). Use of ondansetron during pregnancy and the risk of major congenital malformations: A systematic review and meta-analysis. Reprod. Toxicol. 86, 1–13. 10.1016/j.reprotox.2019.03.001 30849498

[B26] Koren G. (2014). Treating morning sickness in the United States—changes in prescribing are needed. Am. J. Obstet. Gynecol. 211 (6), 602–606. 10.1016/j.ajog.2014.08.017 25151184

[B27] Lavecchia M. Chari R. Campbell S. Ross S. (2018). Ondansetron in pregnancy and the risk of congenital malformations: A systematic review. J. Obstet. Gynaecol. Can. 40 (7), 910–918. 10.1016/j.jogc.2017.10.024 29754832

[B28] Lemon L. S. Bodnar L. M. Garrard W. Venkataramanan R. Platt R. W. Marroquin O. C. (2020). Ondansetron use in the first trimester of pregnancy and the risk of neonatal ventricular septal defect. Int. J. Epidemiol. 49 (2), 648–656. 10.1093/ije/dyz255 31860078 PMC7266534

[B29] Lemon L. S. Zhang H. Hebert M. F. Hankins G. D. Haas D. M. Caritis S. N. (2016). Ondansetron exposure changes in a pregnant woman. Pharmacotherapy 36 (9), e139–41. 10.1002/phar.1796 27374186 PMC5738660

[B30] Lowe S. A. Armstrong G. Beech A. Bowyer L. Grzeskowiak L. Marnoch C. A. (2020). SOMANZ position paper on the management of nausea and vomiting in pregnancy and hyperemesis gravidarum. Aust. N. Z. J. Obstet. Gynaecol. 60 (1), 34–43. 10.1111/ajo.13084 31657004

[B31] Moher D. Liberati A. Tetzlaff J. Altman D. G. PRISMA Group (2009). Preferred reporting items for systematic reviews and meta-analyses: The PRISMA statement. BMJ 339, b2535. 10.1136/bmj.b2535 19622551 PMC2714657

[B32] Özdemirci Ş. Akpınar F. Bilge M. Ozdemirci F. Yilmaz S. Esinler D. (2014). The safety of ondansetron and chlorpromazine for hyperemesis gravidarum in first trimester pregnancy. Gynecol. Obstet. Reprod. Med. 20, 81–84. Corpus ID: 6404132.

[B33] Parker S. E. Van Bennekom C. Anderka M. Mitchell A. A. National Birth Defects Prevention Study (2018). Ondansetron for treatment of nausea and vomiting of pregnancy and the risk of specific birth defects. Obstet. Gynecol. 132 (2), 385–394. 10.1097/AOG.0000000000002679 29995744

[B34] Pasternak B. Svanström H. Hviid A. (2013). Ondansetron in pregnancy and risk of adverse fetal outcomes. N. Engl. J. Med. 368 (9), 814–823. 10.1056/NEJMoa1211035 23445092

[B35] Picot C. Berard A. Grenet G. Ripoche E. Cucherat M. Cottin J. (2020). Risk of malformation after ondansetron in pregnancy: An updated systematic review and meta-analysis. Birth Defects Res. 112 (13), 996–1013. 10.1002/bdr2.1705 32420702

[B36] Raymond S. H. (2013). A survey of prescribing for the management of nausea and vomiting in pregnancy in Australasia. Aust. N. Z. J. Obstet. Gynaecol. 53 (4), 358–362. 10.1111/ajo.12045 23346891

[B37] Sakran R. Shechtman S. Arnon J. Diav-Citrin O. (2021). Pregnancy outcome following *in-utero* exposure to ondansetron: A prospective comparative observational study. Reprod. Toxicol. 99, 9–14. 10.1016/j.reprotox.2020.11.005 33212170

[B38] Shehmar M. Ma M. Nelson-Piercy C. Gadsby R. (2016) The management of nausea and vomiting of pregnancy and hyperemesis gravidarum (Green-top guideline No. 69). United Kingdom: Royal College of Obstetricians & Gynaecologists. Available at: https://www.rcog.org.uk/guidance/browse-all-guidance/green-top-guidelines/the-management-of-nausea-and-vomiting-of-pregnancy-and-hyperemesis-gravidarum-green-top-guideline-no-69/ .

[B39] Stang A. (2010). Critical evaluation of the Newcastle-Ottawa scale for the assessment of the quality of nonrandomized studies in meta-analyses. Eur. J. Epidemiol. 25 (9), 603–605. 10.1007/s10654-010-9491-z 20652370

[B40] Suarez E. A. Boggess K. Engel S. M. Sturmer T. Lund J. L. Funk M. J. (2021). Ondansetron use in early pregnancy and the risk of late pregnancy outcomes. Pharmacoepidemiol. Drug Saf. 30 (2), 114–125. 10.1002/pds.5151 33067868

[B41] Taylor L. G. Bird S. T. Sahin L. Tassinari M. S. Greene P. Reichman M. E. (2017). Antiemetic use among pregnant women in the United States: The escalating use of ondansetron. Pharmacoepidemiol. Drug Saf. 26 (5), 592–596. 10.1002/pds.4185 28220993

[B42] van Gelder M. M. H. J. Nordeng H. (2021). Antiemetic prescription fills in pregnancy: A drug utilization study among 762, 437 pregnancies in Norway. Clin. Epidemiol. 13, 161–174. 10.2147/CLEP.S287892 33664595 PMC7924249

[B43] Wells G. A. Shea B. O'Connell D. Peterson J. Welch V. Losos M. (2001). The newcastle-ottawa scale (NOS) for assessing the quality of non randomised studies in meta-analyses. Canada: The Ottawa Hospital. Available at: https://www.ohri.ca//programs/clinical_epidemiology/oxford.asp .

[B44] Werler M. M. Yazdy M. M. Kasser J. R. Mahan S. T. Meyer R. E. Anderka M. (2014). Medication use in pregnancy in relation to the risk of isolated clubfoot in offspring. Am. J. Epidemiol. 180 (1), 86–93. 10.1093/aje/kwu096 24824985 PMC4133556

[B45] Zambelli-Weiner A. Via C. Yuen M. Weiner D. J. Kirby R. S. (2019). First trimester ondansetron exposure and risk of structural birth defects. Reprod. Toxicol. 83, 14–20. 10.1016/j.reprotox.2018.10.010 30385129

