# Supplementary material for: Risk of abnormal pregnancy outcomes after using ondansetron during pregnancy: A systematic review and meta-analysis
Source: Front Pharmacol. 2022 Sep 2;13:951072. doi: 10.3389/fphar.2022.951072 (PMC9480102; doi:10.3389/fphar.2022.951072)
Supplement: Supplementary file 1 [file Table1.DOCX]

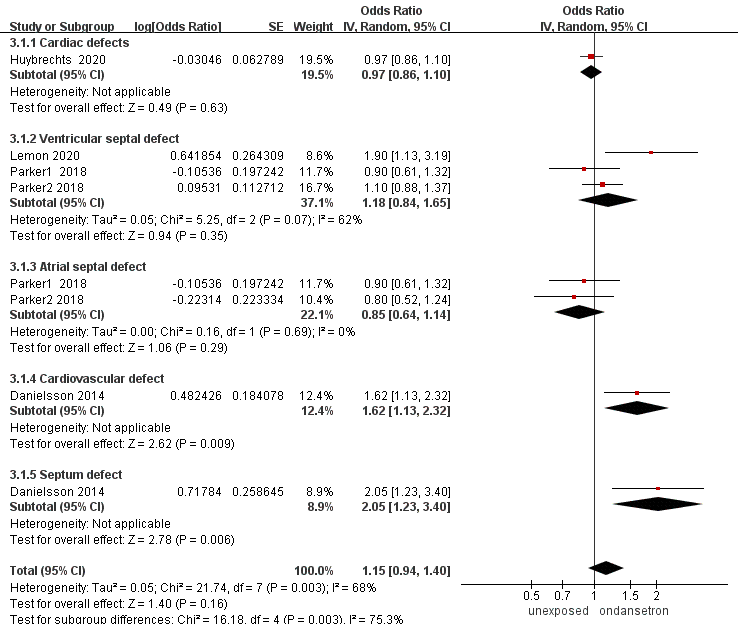


Fig S1 Sensitivity analysis result of cardiac defects


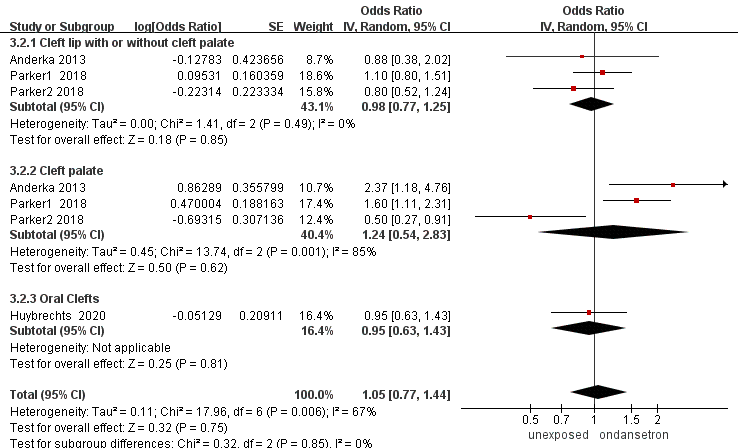


Fig S2 Sensitivity analysis result of orofacial clefts


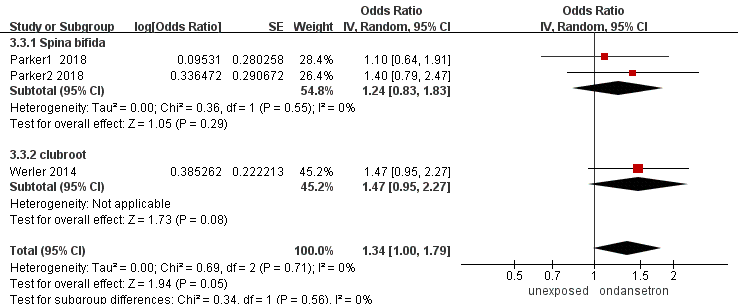


Fig S3 Sensitivity analysis result of spinal limb defects


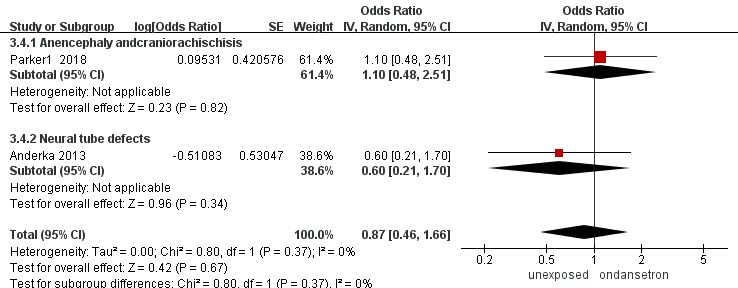


Fig S4 Sensitivity analysis of neural tube defects


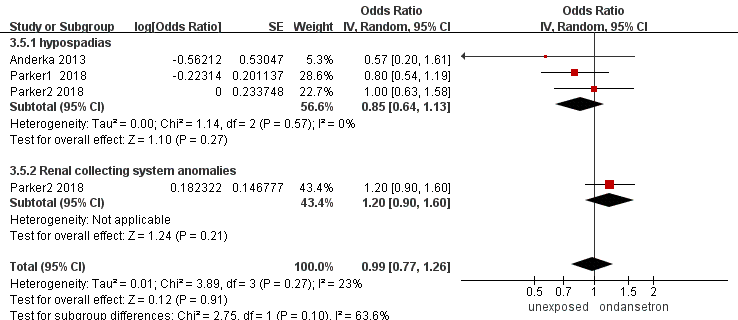


Fig S5 Sensitivity analysis of urinary tract deformities

**Supplementary**

**Table S1 Search Strategy**

| # | 检索 | 结果 |
| --- | --- | --- |
| PubMed | | |
| 1 | (ondansetron[Title/Abstract]) OR (ondansetron[MeSH Terms]) | 5,125 |
| 2 | (((Pregnancy Outcome[MeSH Terms]) OR (abnormalities[MeSH Terms])) OR (Infant, Newborn[MeSH Terms])) OR (Delivery, Obstetric[MeSH Terms]) | 1,273,044 |
| 3 | ((((pregnant[Title/Abstract]) OR (newborn[Title/Abstract])) OR (pregnancy[Title/Abstract])) OR (perinatal[Title/Abstract])) OR (delivery[Title/Abstract]) | 1,131,165 |
| 4 | #2 OR #3 | 2,121,469 |
| 5 | #1 AND #4 | 422 |
| The Cochrane Library | | |
| 1 | MeSH descriptor: [ondansetron] explode all trees | 1,363 |
| 2 | (ondansetron):ti,ab,kw | 3,861 |
| 3 | #1 OR #2 | 3,861 |
| 4 | MeSH descriptor: [Pregnancy Outcome] explode all trees | 3,844 |
| 5 | MeSH descriptor: [Infant, Newborn] explode all trees | 16,999 |
| 6 | (pregnant):ti,ab,kw OR (newborn):ti,ab,kw OR (pregnancy):ti,ab,kw OR (perinatal):ti,ab,kw OR (Obstetric):ti,ab,kw" | 92,409 |
| 7 | #4 OR #5 OR #6 | 92,546 |
| 8 | #3 AND #7 | 285 Trial |
| Embase | | |
| 1 | Ondansetron:ti,ab,kw | 7,105 |
| 2 | pregnant:ti,ab,kw OR pregnancy:ti,ab,kw OR perinatal:ti,ab,kw OR birth:ti,ab,kw OR childbirth:ti,ab,kw OR delivery:ti,ab,kw OR obesity:ti,ab,kw OR 'maternal fetal':ti,ab,kw | 1,987,090 |
| 3 | #1 AND #2 | 555 |
| CNKI | | |
| 1 | SU=昂丹司琼 OR TKA=昂丹司琼 | 1,848 |
| 2 | SU=孕妇 OR SU=产妇 OR SU=新生儿 OR SU=胎儿 OR SU=妊娠 OR SU=母体 OR SU=分娩 OR SU=围产 OR TKA=孕妇 OR TKA=产妇 OR TKA=新生儿 OR TKA=胎儿 OR TKA=妊娠 OR TKA=母体 OR TKA=分娩 OR TKA=围产 | 968,786 |
| 3 | #1 AND #2 | 90 |
| CBM | | |
| 1 | "昂丹司琼"[常用字段:智能] | 2534 |
| 2 | "孕妇"[常用字段:智能] OR "产妇"[常用字段:智能] OR "新生儿"[常用字段:智能] OR "胎儿"[常用字段:智能] OR "妊娠"[常用字段:智能] OR "母体"[常用字段:智能] OR "分娩"[常用字段:智能] OR "围产期"[常用字段:智能] | 608,994 |
| 3 | #1 AND #2 | 86 |
| VIP | | |
| 1 | M=昂丹司琼 | 718 |
| 2 | M=孕妇 OR M=产妇 OR M=新生儿 OR M=胎儿 OR M=妊娠 OR M=母体 OR M=分娩 OR M=围产 | 447,282 |
| 3 | #1 AND #2 AND #3 | 14 |
| Wan Fang DATA | | |
| 1 | 题名或关键词=昂丹司琼 OR 主题=昂丹司琼 | 1930 |
| 2 | 题名或关键词=孕妇 OR 题名或关键词=产妇 OR 题名或关键词=新生儿 OR 题名或关键词=胎儿 OR 题名或关键词=妊娠 OR 题名或关键词=母体 OR 题名或关键词=分娩 OR 题名或关键词=围产 OR主题=孕妇 OR 主题=产妇 OR 主题=新生儿 OR 主题=胎儿 OR 主题=妊娠 OR 主题=母体 OR 主题=分娩 OR 主题=围产 | 2,450,128 |
| 3 | #1 AND #2 | 106 |

**Table S2 Excluding studies**

| Author year | Action | Reason |
| --- | --- | --- |
| Reichmann 2016 | Exclude | Wrong design |
| DAMKIER 2020 | Exclude | Wrong design |
| KIRBY 2020 | Exclude | Wrong design |
| NA 2013 | Exclude | Wrong design |
| Lavecchia 2019 | Exclude | Wrong design |
| Haas 2018 | Exclude | Wrong design |
| Collins 2019 | Exclude | Wrong design |
| Siminerio 2016 | Exclude | Wrong design |
| Siu 2006 | Exclude | Wrong design |
| Koren 2014 | Exclude | Wrong design |
| Meng M 2013 | Exclude | Wrong design |
| Lavecchia 2018 | Exclude | Wrong design |
| NR 2016 | Exclude | Wrong design |
| Margaret 2014 | Exclude | Wrong exposure |
| Werler 2014 | Exclude | Wrong exposure |
| Fejzo 2015 | Exclude | Wrong outcome |
| Schrager 2019 | Exclude | Wrong outcome |
| Huybrechts 2019 | Exclude | Old research |
| Anderka 2012 | Exclude | Old research |

**Table S3 The risk of bias of the including studies**

| Aouthor Year | Study design | Is the Case Definition Adequate /Representativeness of the Exposed Cohort | Representativeness of the Cases /Selection of the Non-Exposed Cohort | Selection of Controls /Ascertainment of Exposure | Definition of Controls /Demonstration That Outcome of Interest Was Not Present at Start of Study | Study controls for Age | Study controls for any additional factor /Comparability of Cohorts on the Basis of the Design or Analysis | Ascertainment of Exposure /Assessment of Outcome | Same method of ascertainment for cases and controls /Was Follow-Up Long Enough for Outcomes to Occur | Non-Response Rate /Adequacy of Follow Up of Cohorts | Total score |
| --- | --- | --- | --- | --- | --- | --- | --- | --- | --- | --- | --- |
| Anderka,2013 | Cohort study | 1 | 1 | 1 | 1 | 0 | 0 | 1 | 1 | 1 | 7 |
| Asker,2005 | Cohort study | 1 | 1 | 1 | 1 | 0 | 0 | 0 | 1 | 1 | 6 |
| Berard,2019 | Cohort study | 1 | 1 | 1 | 1 | 0 | 0 | 1 | 1 | 1 | 7 |
| Colvin,2013 | Cohort study | 1 | 1 | 0 | 1 | 0 | 0 | 1 | 1 | 1 | 6 |
| Danielsson,2014 | Case–control study | 1 | 1 | 1 | 1 | 0 | 0 | 1 | 1 | 1 | 7 |
| Dormuth,2021 | Cohort study | 1 | 1 | 1 | 1 | 0 | 0 | 1 | 1 | 1 | 7 |
| Einarson,2004 | Cohort study | 1 | 1 | 0 | 1 | 0 | 0 | 0 | 1 | 1 | 5 |
| Fejzo,2016 | Cohort study | 1 | 1 | 0 | 1 | 0 | 1 | 0 | 1 | 1 | 6 |
| Huybrechts, 2020 | Cohort study | 1 | 1 | 1 | 1 | 0 | 0 | 1 | 1 | 1 | 7 |
| Lemon,2020 | Cohort study | 1 | 1 | 1 | 1 | 0 | 0 | 1 | 1 | 1 | 7 |
| ÖZDEMİRCİ,2014 | Cohort study | 1 | 1 | 1 | 1 | 0 | 0 | 0 | 1 | 1 | 6 |
| Parker, 2018 | Case–control | 1 | 1 | 1 | 1 | 0 | 0 | 0 | 1 | 1 | 6 |
| Pasternak, 2013 | Cohort study | 1 | 1 | 1 | 1 | 0 | 0 | 1 | 1 | 1 | 7 |
| Sakran,2021 | Case-control study | 1 | 1 | 1 | 1 | 1 | 1 | 0 | 1 | 1 | 8 |
| Suarez,2020 | Cohort study | 1 | 1 | 1 | 1 | 0 | 0 | 1 | 1 | 0 | 6 |
| Zambelli-Zambelli-Weiner,2019 | Case-control study | 1 | 1 | 1 | 1 | 1 | 1 | 1 | 1 | 1 | 9 |

**Table S4 Details on variables for which studies adjusted**

| Study | Adjusted variables |
| --- | --- |
| Fejzo  2016 [33] | ethnicity, education, termination, miscarriage, etc. |
| ÖZDEMİRC 2014 [34] | NR |
| Colvin 2013 [35] | gestational age, smoking during pregnancy, SEIFA, sex, and parity. |
| Berard 2019 [36] | maternal comorbidities in the year before 1DG，Use of health services in the year before 1DG. |
| Asker 2005 [37] | year of birth, maternal age, parity, smoking, and unwanted childlessness. |
| Dormuth 2021 [22] | calendar year of pregnancy outcome, maternal age, pregnancy history (live births, spontaneous abortions, induced abortions, and stillbirths in the previous 5 years), hospitalization for  hyperemesis gravidarum, history of diabetes, immunodeficiency disorders, prior health service use, and claims for prescription drugs suspected to influence risk for adverse pregnancy outcomes. |
| Lemon 2020 [23] | nausea/vomiting/hyperemesis/gastrointestinal infection, total encounters, insurance type, maternal age, race, years of edu-  cation, marital status, pre-gravid BMI, parity, history of termination/miscarriage/abortion, drug use, arrhythmia, chronic hypertension, collagen vascular disease,  lower gastrointestinal disease, upper gastrointestinal disease, diabetes (any), maternal structural heart disease, ultrasound with nuchal fold measurement in preg-  nancy, smoking status and patient of Magee-resident clinic. |
| Einarson 2004 [38] | NR |
| Huybrechts 2020 [24] | Maternal conditions,Concomitant medications,treatment indication (nausea and vomiting in pregnancy, hyperemesis gravidarum) and associated conditions (weight loss, electrolyte and laboratory abnormalities, dehydration, gastroesophageal reflux), state of residence, maternal chronic conditions (kidney disease, Crohn disease, irritable bowel syndrome, ulcerative colitis, overweight or obesity, underweight), concomitant medications (anticonvulsants, triptans, progestins, corticosteroids), and general markers of comorbid illness and disease severity (number of prescriptions for medications other than antiemetics, number of distinct diagnoses, number of outpatient visits, hospitalizations, and emergency department visits). Maternal conditions and concomitant medication use were measured from 3 months before the start of pregnancy through the end of the first trimester. General markers of the burden of illness were measured during the 3 months before pregnancy because these  measures may be affected by early pregnancy complications. |
| Suarez 2020 [25] | NR |
| Pasternak 2013 [39] | Hospitalization for hyperemesis gravidarum or nausea and vomiting and exposure to antiemetics other than ondansetron within the respective exposure time window. |
| Werler 2014 [40] | study site, first born, sex, body mass index (weight (kg)/height (m)2), and maternal smoking. |
| Weiner 2019 [41] | mother’s age, infant year of birth, and infant gender. |
| Sakran 2021 [21] | maternal age, Previous miscarriage. |
| Danielsson 2014 [42] | year of birth,  maternal age, parity, smoking in early pregnancy, and body mass index. |
| Parker 2018 [43] | maternal age, education, folic acid use, year, and study site. |
| Anderka 2013 [44] | maternal age, race/ethnicity and education, parity, plurality, previous miscarriage, any smoking in the month before conception through the first trimester, body mass index, infant sex, any  folic acid use in the month before conception through the first trimester, use of unknown antiemetic, site, and expected year of delivery. |
| Couse 2020 [45] | NR |
| Lemon 2016 [46] | NR |
| Ferreira 2012 [47] | NR |
